# Retraction: A Strain of an Emerging Indian *Xanthomonas oryzae* pv. oryzae Pathotype Defeats the Rice Bacterial Blight Resistance Gene *xa13* Without Inducing a Clade III *SWEET* Gene and Is Nearly Identical to a Recent Thai Isolate

**DOI:** 10.3389/fmicb.2020.609848

**Published:** 2020-10-21

**Authors:** Frontiers Editorial Office

The journal retracts the 13 November 2018 article cited above.

Experiments performed subsequent to publication, using genotype-verified, single seed-descent IR2R4, IRBB5B, and IRBB1B3 plants, revealed that IX-280 and SK2K2-3, though compatible with xa5a, are not compatible with xa1a3 as originally reported. The discrepancy is postulated to be due to genotype contamination in the seeds used for initial characterization, as several older seed stocks labeled “IRBB1B3,” including stocks used to pathotype these strains originally, when tested were found to be a mix of Xa1a3 and xa1a3 genotypes. The authors present the genome comparisons and TAL effector analysis for IX-280 and SK2-3 in a new manuscript, Carpenter et al., 2020 (new submission: https://www.frontiersin.org/articles/10.3389/fmicb.2020.579504/full).

The authors requested the retraction and sincerely regret any inconvenience this may have caused to the reviewers, editors and readers of Frontiers in Microbiology.

